# Peste des Petits Ruminants (PPR) in Ethiopia: Analysis of a national serological survey

**DOI:** 10.1186/1746-6148-4-34

**Published:** 2008-09-12

**Authors:** Agnès Waret-Szkuta, François Roger, David Chavernac, Laikemariam Yigezu, Geneviève Libeau, Dirk U Pfeiffer, Javier Guitián

**Affiliations:** 1 Epidemiology Division, Department of Veterinary Clinical Sciences, The Royal Veterinary College, Hawkshead Lane, North Mymms, Hatfield, Herts. AL9 7TA, UK; 2 CIRAD, Campus International de Baillarguet, 34 398 Montpellier Cedex 5, France; 3 FAO, Addis Ababa, Ethiopia

## Abstract

**Background:**

Peste des petits ruminants (PPR) is a contagious viral disease of small ruminants in Africa and Asia. In 1999, probably the largest survey on PPR ever conducted in Africa was initiated in Ethiopia where 13 651 serum samples from 7 out of the 11 regions were collected and analyzed by competitive enzyme-linked immunosorbent assay (cELISA). The objective of this paper is to present the results of this survey and discuss their practical implications for PPR-endemic regions.

**Methods:**

We explored the spatial distribution of PPR in Ethiopia and we investigated risk factors for positive serological status. Intracluster correlation coefficients (ρ), were calculated for 43 *wereda *(administrative units).

**Results:**

Seroprevalence was very heterogeneous across regions and even more across *wereda*, with prevalence estimates ranging from 0% to 52.5%. Two groups of *weredas *could be distinguished on the basis of the estimated ρ: a group with very low ρ (ρ < 0.12) and a group with very high ρ (ρ > 0.37).

**Conclusion:**

The results indicate that PPRV circulation has been very heterogeneous, the values for the ρ may reflect the endemic or epidemic presence of the virus or the various degrees of mixing of animals in the different areas and production systems. Age appears as a risk factor for seropositive status, the linear effect seeming to confirm in the field that PPRV is highly immunogenic. Our estimates of intracluster correlation may prove useful in the design of serosurveys in other countries where PPR is of importance.

## Background

Peste des Petits Ruminants (PPR) is a severe and highly infectious viral disease of small ruminants. The PPR virus (PPRV) belongs to the genus *Morbillivirus *in the family Paramyxoviridae. It is closely related to the rinderpest virus of bovines and buffaloes, distemper virus of dogs and other wild carnivores, human measles virus and Morbilliviruses of marine mammals [1-4]. In small ruminants, infection by PPRV is characterized by sudden depression, fever, nasal and ocular discharge, diarrhoea and occasionally death. Morbidity in the range of 10 to 80% and mortality proportions from 0 to 90% have been reported. The wide range of reported values is likely to be influenced by differences between species (sheep or goats), production systems and levels of natural or acquired immunity [5-10].

PPR was first described in West Africa in 1942 [11]. Nowadays the disease is recognized as responsible for mortality and morbidity across most of the sub-Saharan African countries situated north of the equator, in the Arabian Peninsula, in India and in numerous other countries in Asia [6,12-14]. Although nationwide serosurveys have been conducted in countries such as the sultanate of Oman, Turkey, Jordan and India, information on the frequency and distribution of PPR is often lacking when control or eradication campaigns are initiated [15-18]. Control of PPR in endemic areas relies mainly in vaccination [19,20]. In 1989 a homologous vaccine that induces lifelong immunity in both sheep and goats was developed [6,21-23]. The vaccine is innocuous on pregnant sheep and goats at any stage of gestation and induces the production of colostral anti-PPR antibodies that have been found in kids up to 3 months old [6,23].

Ethiopia has the most important livestock population in Africa and is ranked 9^th ^in the world [24]. The livestock sub sector accounts for 40% of the agricultural gross domestic product (GDP) and 20% of the total GDP (Aklilu Y. An audit of livestock marketing status in Kenya, Ethiopia and Sudan. PACE/OUA/IBAR, 2002) without considering the livestock contribution in terms of traction power, fertilizing and mean of transport. Sheep and goat populations are estimated to be 20.7 million and 16.4 million respectively [25]. Sheep and goats contribute 25% of the meat domestically consumed with a production surplus mainly being exported as live animals [26,27]. Both species also contribute 50% of the domestic needs in wool, about 40% of skins and 92% of the value of hides and skin exported [28]. The annual production of sheep and goat meat is estimated as 56 560 and 28 650 tonnes respectively [24]. PPR was clinically suspected for the first time in Ethiopia in 1977 in a goat herd in the Afar region, East of the country [9,29]. Clinical and serological evidence of its presence has been reported by Taylor (1984) and later confirmed in 1991 with cDNA probe in lymph nodes and spleen specimens collected from an outbreak in a holding near Addis Ababa [29]. During the nineties, several small serological surveys were conducted, mainly east of an imaginary line that would run parallel to the Rift valley and pass through Addis Ababa. In 1994 Roger and Bereket (CIRAD-EMVT report n°96006, Montpellier, 1996) found seroprevalences of up to 33% in sheep and 67% in goats near selected urban areas. In 1996 Gelagay found that 14.6% of sheep sampled along 4 roads from Debre Berhan to Addis Ababa were seropositive [30]. In 1997 Yayerade found up to 100% of seropositive individuals in groups of adult male sheep and animals that survived suspected outbreaks. Although these studies provide very limited and potentially biased information about the frequency and distribution of PPR in Ethiopia, they clearly suggest that the virus has been circulating extensively among the small ruminant population of Ethiopia during the nineties. Based on the reported morbidity and mortality of the infection and the size and structure of the small ruminant sector it is likely that PPR became one of the most economically important livestock diseases in the country [12,31].

In 1999, a serological survey on PPR was conducted in Ethiopia with the aim of informing a subsequent vaccination campaign which would be the first large scale vaccination campaign against PPR in the country. As part of the survey, a total of 13 753 sheep and goats were sampled. To our knowledge, this is the largest serological survey on PPR ever conducted in Africa. The objective of this paper is to describe the results of this survey and discuss its practical implications.

## Methods

### Administrative structure and distribution of small ruminants in Ethiopia

The Ethiopian administrative structure has frequently been subject to modification. To date there are 11 Regions or States or *Kelel *composed of 71 zones. These zones include about 546 districts or *wereda *or *woreda*. Each *wereda *is composed of *kebelles *or *Peasant Associations *that are an aggregation of *got*, a *got *being a group of 3 to 5 villages although the difference between *got *and village is sometimes unclear in the field [25]. The very diverse relief of the country determines several geoclimatic zones. The central part is characterized by mountainous massifs and covers half of the territory. It is a zone of Highlands ranging from 2 300 to 3 500 m called *Dega *surrounded by a temperate transition zone between 1 500 and 2 300 m called *Woinadega *that dives in the central Rift Valley towards the south west. East the tectonic deflection opens on the lowland areas *Bereha *and *Kola *(0 to 1 200 m), zones of pastoral nomadic livestock husbandry [32].

In 1995 about three quarters of the sheep stock was located in the Ethiopian highlands (> 1 500 m) [33]. A recent report (Aklilu Y. consultancy report USAID/Ethiopia and EGAT Office of USAID/Washington, 2005) suggests that nowadays around half of the sheep are kept in the highlands and half in the lowlands.

### The 1999 Survey

According to a report of the Ethiopian Ministry of Agriculture and Rural Development of 2005 the serological survey was initiated in 1999 to determine the distribution of PPR across the country and to identify areas of increased risk. Compared to the previous studies the geographical coverage is extended to north and western parts of the country. The objective of the survey was to inform the design of a strategy for cost effective control of the disease. The survey was implemented as a subcomponent of the animal health component under the National Livestock Development Project (NLDP) of the Ethiopian Ministry of Agriculture. This project started in 1999 and was financed by the African Development Bank (ADB). The original plan was to collect 8 000–12 000 sera samples through 7 regional veterinary laboratories located in 7 regional states to inform a 3 year vaccination campaign to be started in 2004. Thus vaccination would be implemented in those *wereda *identified by the regional states as endemic for PPR as well as in the neighbouring *wereda*.

Multistage sampling was the chosen sampling strategy, with 4 hierarchical stages as illustrated in Table 1. The first level of selection was the region; only those regions with a veterinary laboratory (7 out of the 11 regions) were selected. Within each of the selected regions (Afar, Amhara, Benishangul Gumuz, Oromia, SNNPR, Somali, Tigray) *weredas*, *kebelles *and villages were randomly selected (Epidemiology unit, Ministry of Agriculture and Rural Development personal communication). Within each of the selected villages, 20 animals (either sheep or goats) were supposed to be randomly selected but were most probably purposively selected because of field and time constraints although we have not been able to completely ascertain the details of the selection process (Laikemariam Yigezu, former PACE coordinator and head of Microbiology unit at the Sebeta laboratory, personal communication).

**Table 1:** Structure of the different administrative levels of sampling

**Included in the above administrative level**	**Region (Ref)**	***wereda***	***kebelle***	**village**
Total	7	84		
Mode		9	5	5
Average		12.14	4.98*	4.45**
Range		8 – 19	2 – 9	1 – 5

*average number of *kebelle *per *wereda *where *kebelle *level was available

**average number of villages per *kebelle *where *village *level was available

The table shows the 4 administrative levels of sampling in the 1999 national serological survey on PPR in Ethiopia. The first level of selection was the region with 7 regions selected. Within each of the regions *weredas*, *kebelles *and villages were randomly selected. For each level the mode, average and range of units included in the above administrative level are presented.

### Laboratory techniques

Serum samples were analyzed by the National Animal Health Research Center (NAHRC, Sebeta, Ethiopia) using a competitive ELISA kit according to the instructions of the manufacturer (Institute for Animal Health, Pirbright Laboratory, UK). The ELISA micro-plates were read with an immunoskan reader (Flow laboratories, UK) with an inference filter of 492 nm. The reader was connected to a computer loaded with ELISA Data Information (EDI) software (FAO/IAEA, Vienna, Austria), which was used to automate the reading and calculation of the percentage of inhibition (PI) values. The OD (Optical Density) values were converted to percentage inhibition using the following formula:

PI = 100 - (OD control or test serum/OD monoclonal control)*100

The samples with PI > 50% (cut-off) were considered as positives.

### Data management

The data were entered and stored electronically in Microsoft Office Access 2003. The fields included in the database are presented in Table 2. Laboratory results and field information collected during the sampling were entered into the database. When age was given as a binary variable (young vs. adult; n = 157 entries), it was considered to be a missing value. When age was given as number of months or years of age (values ranging from 6 months to 10 years old; n = 465) the original variable was recategorized into 4 categories (< 1; 1 < 2; 2 < 3; > = 3) to match with the categories given for the remaining 4 181 animals for which age was given using these four categories.

**Table 2:** List of relevant variables included in the database along with the number of observations available

**Variable**	**Number of samples for which it was recorded**	**% of samples for which it was recorded**
**Localisation**		
Region	13 651	100
*Wereda*	13 613	99.7
*Kebelle*	9 328	68.3
Village	980	7.2

**Species^a^**	13 651	100
**Age^a^**	4 648	34
**Sex^a^**	5 868	43

**Results**		
OD	13 651	100
PI	13 651	100
Interpretation	13 651	100

a: categories defined in Table 4

For each variable recorded during the 1999 serological survey on PPR in Ethiopia and stored in the database, the number of samples for which information was recorded and the % out of the total number of samples it represents are shown.

### Data analysis and spatial description

Descriptive statistics of the studied variables were obtained. Within each *wereda*, the following parameters were obtained:

- seroprevalence (number of positive valid samples/number of individuals sampled in the *wereda*)

- intra-*kebelle *correlation coefficient (ρ) for the 43 *weredas *for which information about the *kebelle *of origin of the samples was available calculated as:

$$\rho =\frac{\sigma^{2}(b)}{\sigma^{2}(b)+\sigma^{2}(w)}$$

Where:

$$\text{Between-group variance}:\sigma^{2}(b)=\frac{\sum_{i=1}^{K}n_{i}{\left({\bar{x}}_{i}-\bar{x}\right)}^{2}}{\left(K-1\right)}$$

$$\text{Within-group variance}:\sigma^{2}(w)=\frac{\sum_{i=1}^{K}\sum_{j=1}^{n_{i}}{\left(x_{ij}-{\bar{x}}_{i}\right)}^{2}}{\left(n-K\right)}$$

For a *wereda *in which *K kebelle*s are sampled with *n*_*i *_samples obtained from *kebelle i*

Chloropleth maps were produced using ArcGIS version 9.1 (ESRI, Redlands, California) to show the distributions of i) seroprevalence by *wereda *and ii) intra-*kebelle *correlation coefficient.

The hypotheses that species, age group and sex significantly differed between positive and negative animals were first tested in a univariate analysis by means of 2-tailed chi-squared tests without adjustment for clustering of observations within *wereda*. In a second step, a logistic regression model was used to assess the association between the potential risk factors sex, age and species and the outcome variable PPR serological status. The three independent variables were forced into the model. *Wereda *was included as a random effect to account for clustering within *weredas*. In the multivariate analysis, the first two age categories (less than 1 year and between 1 and 2 years) were collapsed into a single category due to the low numbers of observations in the "< 1 year" group. Associations were deemed significant when P ≤ 0.05 by Wald test. The reliability of the regression coefficient estimates was assessed by testing the sensitivity of the quadrature approximation.

To assess whether the intra-cluster correlation was associated with the magnitude of the serological response of the animals in the *wereda*, we calculated non-parametric correlations (Spearman) between ρ and the 50^th^, 75^th ^and 90^th ^percentiles of the ELISA inhibition percentage and between ρ and the seroprevalence.

Statistical analyses were conducted using SPSS 15.0 for Windows^® ^(SPSS Inc., Chicago, Illinois) and Stata 9.0 (Stata Corporation, College Station, TX).

## Results

### Seroprevalence of PPR in Ethiopia

One hundred and two individual observations were dropped because of missing serological results. The remaining 13 651 individual observations were used in the analysis. The variables included in the dataset and the number of observations for which each variable was available are presented in Table 2. The periods of sampling, submission and analysis, based on the samples for which dates were available, lasted from 26 March 1999 to 5 June 2002, 19 October 1999 to August 2002 and 6 February 2001 to 13 February 2003, respectively.

The distribution of samples across regions and the prevalence per region are presented in Table 3.

**Table 3:** Prevalence of PPR in the seven surveyed regions

**Regions**	**Number of samples collected in each region and % of the whole survey**	**Prevalence with 95% Confidence Intervals**
Afar	1653 (12.1%)	15.3% (13.6–17.0)
Amhara	5992 (43.9%)	4.6% (4.0–5.1)
Benishangul Gumuz	729 (5.3%)	8.0% (6.0–9.9)
Oromia	2290 (16.8%)	1.7% (1.2–2.2)
SNNPR	1622 (11.9%)	1.8% (1.1–2.4)
Somali	465 (3.4%)	21.3% (17.6–25.0)
Tigray	900 (6.6%)	15.3% (13.6–15.9)
Total	13651 (100%)	6.4% (6.0–6.8)

Number of samples collected and prevalence of PPR in each of the surveyed regions. In brackets: % of the whole survey they represent and 95% confidence intervals.

There were large variations between the different regions in the distribution of the samples. Most of the samples have been collected in the northern part of the country and particularly in the Amhara region (43.9% of total samples collected). In regions like Somali or Afar there are about the same numbers of samples collected per *wereda *whereas in Amhara differences across *wereda *are more pronounced perhaps as a result of accessibility constraints. Similarly, few samples were collected in 6 *weredas *of Tigray region that had never been surveyed before.

**Figure 1:**
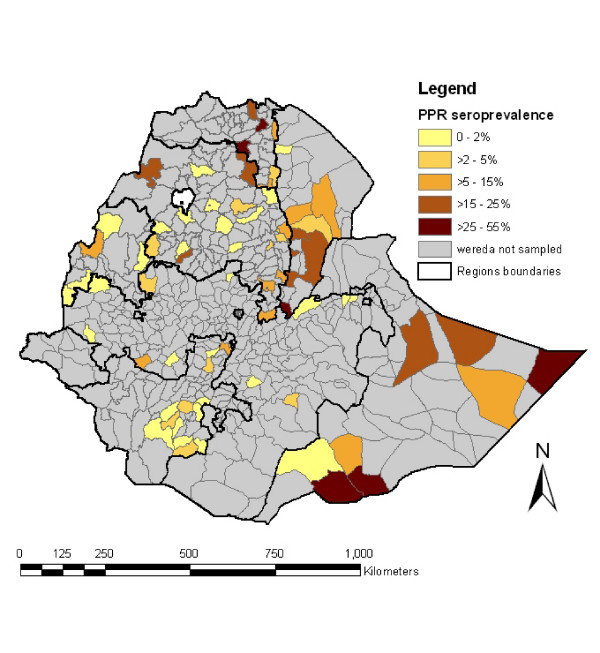
**Seroprevalence of PPR across wereda in Ethiopia**. Administrative map of Ethiopia indicating the regions and *weredas *boundaries. For each *wereda *seroprevalence of PPR was calculated by dividing the number of positive valid samples by the number of individual sampled in the wereda. As the colour gets browner higher is the seroprevalence found in the area. In grey, wereda for which no data was available.

There were important differences in the prevalence across regions, with the Oromia region showing the lowest prevalence (1.7%, 95% CI: 1.2–2.9) and the Somali region the highest (21.3%, 95% CI: 17.6–38.8) (Table 3). The variations are even more important for prevalence at the *wereda *level as shown in Figure 1. *Wereda *level prevalence estimates ranged from 0% for Guba in Benishangul region or Ab Ala in Afar region to 52.5% for Dolo Odo in Somali region. *Wereda *with the higher prevalence levels seem to be mainly those in areas of low altitudes where pastoral management systems prevail over sedentary ones.

### Risk factors for positive serological status against PPR

Descriptive statistics for the variables under study and the results of univariate comparisons are presented in Table 4. The proportions of seropositive animals significantly differ between species, age groups and sex categories. In the univariate analysis, sheep were 4.4 more likely and goats 5 times more likely to be seropositive for PPR than shoats (category combining both sheep and goats). Females were 1.3 times more likely to be seropositive than males. Regarding the age of the animals, none of the 41 animals younger than 1 year were positive. The highest prevalence was observed among animals older than 3 years, 12.6% of which were seropositive.

**Table 4:** Descriptive statistics of qualitative variables and univariate associations with seropositive status against PPR (two-tailed *P*-values for the χ^2 ^– test of association).

**Variable**	**Description**	**N**	**% of factor**	**% positive**	**P**
**Species**		13 651	100		< 0.001
	Sheep	4 211	31	8.3	
	Goats	4 585	33.5	9.4	
	Shoats*	4 855	35.5	1.9	
	Not available	1 000	10		

**Age**		4 648	34		0.003
	Under 1 year old	41	0.9	0	
	Between 1 and 2 years old	392	8.4	10.5	
	Between 2 and 3 years old	2 014	43.3	9.7	
	Over 3 years old	2 201	47.4	12.6	
	Not available	9 003	66		

**Sex**		5 868	43		0.013
	Males	1 007	17.2	7.0	
	Females	4 861	82.8	9.4	
	Not available	7 783	57		

* Sheep and goats not being distinguished

Results of 2-tailed chi-squared tests of the hypothesis that species, age group and sex differed between positive and negative animals. A description of each variable is presented including the number and % of each category in the sampled population and the % of positive. The proportions of seropositive animals significantly differ between species, age groups and sex categories (P-value (P) < 0.05).

Results of the logistic regression assessing the relationship between species, age and sex and serological status are presented in Table 5. The only factor significantly associated with the odds of positive serological status was the age of the animal. Increasing age was associated with an increasing odds of seropositive status, with animals over 3 years old having almost twice the odds of been positive than animals under 2 years old.

**Table 5:** Results of a logistic regression of sex, age and species on serological status against PPR with *wereda *as random effect.

**Variable**	**OR**	**P**	**95%CI**
**Sex**			
male	ref		
female	1.15	0.37	0.85–1.54

**Age**			
< 2	ref		
2 – 3	1.27	0.23	0.86–1.88
> 3	1.78	< 0.01	1.20–2.62

**Species**			
sheep	ref		
goat	1.08	0.50	0.86–1.35
shoat	1.42	0.51	0.50–4.04

Results of the logistic regression model used to assess the association between the potential risk factors sex, age and species and the outcome variable PPR serological status. *Wereda *is included as a random effect to account for clustering within *weredas*. The only factor significantly associated with the odds of positive serological status is the age of the animal (P < 0.01). Animals over 3 years old have almost twice the odds of been positive than animals under 2 years old.

As expected, there was strong evidence of significant intra-*wereda *correlation (intra-cluster correlation ρ = 0.36; P < 0.001). Despite the large number of observations per *wereda *(average of 166 animals) and the large intra-*wereda *correlation there was no evidence of unreliability of the quadrature approximation when estimates obtained using different numbers of cutpoints were compared.

### Intracluster correlation coefficient (ρ)

**Figure 2:**
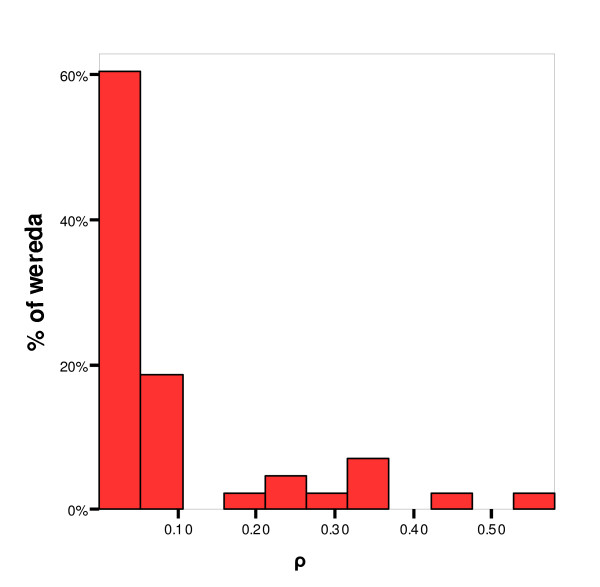
**Distribution of the correlation coefficient (ρ) across wereda**. Histogram showing the values of the intra-cluster correlation coefficient calculated for 43 *weredas *for which information about the *kebelle *of origin of the samples was available as indicated in the Methods section. Two groups can be distinguished: one including almost 80% of the *weredas *with low values of ρ (ρ < 0.12) and the other with 9.3% of the *weredas *showing a strong intracluster correlation (ρ > 0.37).

The 43 *weredas *for which the intracluster correlation coefficient (ρ) was calculated included between 2 and 9 *kebelles *each (median = 5) and these *kebelles *included between 15 and 180 individual animals each (median = 40). The estimated intra-cluster correlation coefficients are presented in Figure 2. Median ρ was 0.029. Although the values seemed very heterogeneous, two groups can be clearly distinguished: One including nearly 80% of the *weredas *(34/43) with very low values of ρ (ρ < 0.12) and the other with 9.3% (4/43) of the *weredas *showing a strong intracluster correlation, ρ > 0.37.

**Figure 3:**
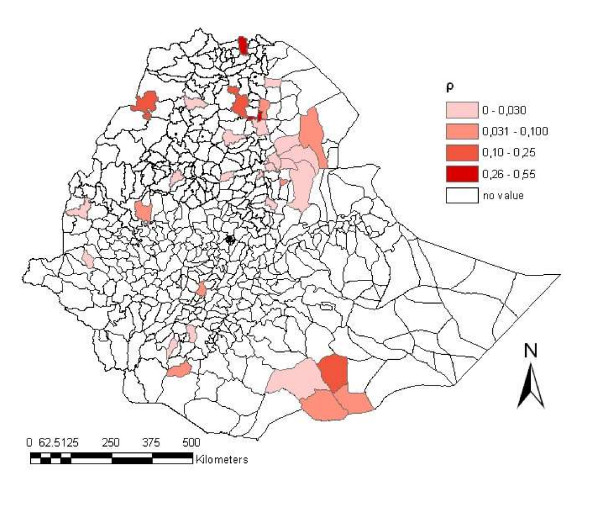
**Distribution of wereda ρ values across Ethiopia in 1999**. Geographical distribution of the values for the intra-cluster correlation coefficient (ρ) by *wereda*. The red is more intense in the *weredas *with a higher value for ρ.

Figure 3 shows the geographical distribution of the values for ρ by *wereda*. The map shows higher values for the correlation coefficient in the north of the country. The intra-*kebelle *correlation coefficient was highly correlated with the inhibition percentages among the animals sampled in the *wereda*: Spearman rank correlation coefficients of 0.45 (P = 0.003) with the median inhibition percentage, 0.45 (P = 0.002) with the 75^th ^percentile of the inhibition percentage and 0.61 (P < 0.001) with the 90^th ^percentile of the inhibition percentage. The intra-*kebelle *correlation coefficient was also highly correlated with the sero-prevalence found for each *wereda*: Spearman rank correlation coefficient of 0.67 (P < 0.001).

## Discussion

Although estimates have to be interpreted with caution because it had not been possible to ensure that random selection was used at all sampling stages, the results indicate that PPR has been circulating in most of the country before large vaccination campaigns were implemented. Lack of large scale vaccination campaigns before the survey was conducted suggests that our seroprevalence estimates are likely to reflect infection [31]. Given the sensitivity and specificity of the test our results are likely to overestimate, slightly, the true proportion of seropositive animals [34,35]. On the other hand, by using the results of an imperfect test as indicators of true infection we are probably underestimating the true values of rho [36]. Despite an overall frequency of 6.4%, the seroprevalence of PPR was above 50% in some *weredas*. PPRV circulation before 2000 has been heterogeneous: areas of low altitudes appear to have suffered more from infection than areas of highlands. Reasons for this may be related to different production systems with exchanges and movements in areas of lowlands being more frequent and involving larger numbers of animals. In Ethiopia small ruminants mainly thrive on free range pasture lands, shrubs and forest grounds. Agro climatic conditions influence the availability of these resources and the movement of animals becomes necessary in order to ensure the provision of fodder and water. This is particularly important during the dry season and in low altitude areas where resources are scarce. In addition, animals are exchanged between households and flocks as a result of social practices and changes in economic conditions that exhibit seasonal patterns. The seasonality of animal movements could partly explain the occurrence of the disease in Ethiopia mainly between the months of March and June [7,17,31].

Although the overall seroprevalence of PPR in Ethiopia appears to be low compared, for example, to the 22.4% reported in Turkey and 33% in India, it is difficult to draw any conclusions because of the differences in sampling procedures in the different studies that affect their representativeness [16,17]. However, a common feature described by the respective authors are heterogeneities, possibly related to agro-climatic and socioeconomic factors.

Age appears to be a risk factor for seropositive status, and its linear effect suggests that PPRV is highly immunogenic, naturally infected animals remaining positive for a long time.

The intra-*kebelle *correlation coefficient was found to be very low in most *wereda *with a small number of them showing high values. Differences in biological factors probably explain this variability. One hypothesis could be the past or recent circulation of PPRV reflected by a low or a high value of ρ, respectively, along with a low or high seroprevalence. Assuming that a high inhibition percentage could reflect recent infection, the strong correlation between ρ and inhibition percentage would be consistent with the interpretation of high ρ as being indicative of current or epidemic presence of the virus in a few *kebelles *within the *wereda*. This correlation then diminishes with time, diluting itself in a *wereda *as a consequence of a relatively rapid turnover of small ruminants (3 years), PPRV being highly immunogenic and that these are serological results. Considering that PPR is as a highly contagious disease and that both the within-and between-*kebelle *spread of infection determine ρ, the low value in certain *weredas *could also be attributed to *weredas *where animals of different *kebelles *mix a lot at market points or at water sources. The absence of an obvious spatial pattern in the distribution of ρ may reflect that spread of the disease has mainly occurred within individual *wereda *as opposed to large scale outbreaks involving several contiguous *wereda*.

Probabilistic sampling is a challenging task in a country with an infrastructure such as Ethiopia, since large areas have to be covered which are not easily accessible. Moreover, sampling frames of lower administrative units are often not available at central level. Under these circumstances, multi-stage sampling strategies such as the one used in the current study are the best option. Despite the random selection of *weredas *within regions, *kebeles *within *weredas *and villages within *kebeles *there is still potential for bias influencing our results due to non-random selection of regions and animals within villages. Although the large variation of values of ρ highlights the limitation of using a summary measure of ρ for a whole country as a basis for a sampling design, our estimates can inform the design effects needed to adjust for cluster sampling in future surveys on PPR in regions with similar production systems. As an example, if we consider the median ρ = 0,029, the standard sample size calculations using simple random sampling with 95% confidence interval for an estimated prevalence of 5% and an accepted error of 1% needs to be inflated by a factor of: D = 1 + 0,029(n-1), n being the average cluster size and D the design effect. That accommodates for the lack of independence between small ruminants belonging to a given *kebelle *[37].

Thus, to design a seroprevalence study at *wereda *level in Ethiopia, the clustering effect of the *kebelles *implies the sample size has to be increased by a factor of 1.26 if 10 small ruminants are to be sampled per *kebelle*, 1.55 if 20 small ruminants are to be sampled, 2.42 if 50 are sampled and 3.87 if a hundred small ruminants are to be sampled in each selected *kebelle*. Our findings are in agreement with other published values for ρ and D related to viral diseases or vector-borne infections. Thus the majority of ρ reported lay below 0.20 with widely varying estimates for highly contagious viral infections as Infectious bovine rhinotracheitis (IBR) [38]. To our knowledge no specific reference was available until now for peste des petits ruminants.

## Conclusion

Our study shows that PPR has extensively circulated across Ethiopia, but that there is large variation between regions and *weredas*. Although in most *weredas *there is a small variation between *kebelles *in some of them there are large differences that indicate the virus has only been introduced recently among some *kebelles *of the *wereda*, if our interpretation of high intracluster correlation coefficients as indicative of more recent introduction is valid, PPRV has been more recently circulating in the North of the country. The results of our study indicate that further research is needed to investigate the association of the presence of disease with the management practices in place. These findings are also important to direct future studies in other countries where PPR is of importance.

## Abbreviations

ADB: African Development Bank; cDNA: complementary DeoxyriboNucleic Acid; cELISA: competitive Enzyme-Linked Immunosorbent Assay; EDI: ELISA Data Information; FAO: Food and Agriculture Organization; GDP: Gross Domestic Product; IAEA: International Atomic Energy Agency; IBR: Infectious Bovine Rhinotracheitis; NAHRC: National Animal Health Research Center; NLDP: National Livestock Development Project; OD: Optical Density; PI: Percentage Inhibition; PPR: Peste des Petits Ruminants; PPRV: Peste des Petits Ruminants virus; ρ: intracluster correlation coefficient.

## Authors' contributions

AWS, DC and LMY collected the data, created the electronic database and cleaned and processed the data for analysis. AWS and JG conceived and performed the data analysis. AWS drafted the manuscript assisted by JG. FR, DUP and GL raised funding for the study and assisted its coordination. All authors helped with the interpretation of the results and read and approved the final manuscript.
